# Role of Platelet Mitochondria: Life in a Nucleus-Free Zone

**DOI:** 10.3389/fcvm.2019.00153

**Published:** 2019-10-29

**Authors:** Hannah Melchinger, Kanika Jain, Tarun Tyagi, John Hwa

**Affiliations:** Department of Internal Medicine, Yale Cardiovascular Research Center, Yale School of Medicine, New Haven, CT, United States

**Keywords:** platelets, mitochondria, anucleate cells, metabolism, apoptosis

## Abstract

Platelets are abundant, small, anucleate circulating cells, serving many emerging pathophysiological roles beyond hemostasis; including active critical roles in thrombosis, injury response, and immunoregulation. In the absence of genomic DNA transcriptional regulation (no nucleus), platelets require strategic prepackaging of all the needed RNA and organelles from megakaryocytes, to sense stress (e.g., hyperglycemia), to protect themselves from stress (e.g., mitophagy), and to communicate a stress response to other cells (e.g., granule and microparticle release). Distinct from avian thrombocytes that have a nucleus, the absence of a nucleus allows the mammalian platelet to maintain its small size, permits morphological flexibility, and may improve speed and efficiency of protein expression in response to stress. In the absence of a nucleus, platelet lifespan of 7–10 days, is largely determined by the mitochondria. The packaging of 5–8 mitochondria is critical in aerobic respiration and yielding metabolic substrates needed for function and survival. Mitochondria damage or dysfunction, as observed with several disease processes, results in greatly attenuated platelet survival and increased risk for thrombovascular events. Here we provide insights into the emerging roles of platelets despite the lack of a nucleus, and the key role played by mitochondria in platelet function and survival both in health and disease.

## Platelet Discovery and Origins

Platelets are small (2–4 μm), short-lived (7–10 days), anucleate circulating cells primarily responsible for the prevention of bleeding and the maintenance of hemostasis (1, 2). A healthy adult has a counts in the range of 150,000–450,000 platelets per microliter of blood, though these counts vary with age and health (3). Platelets were first identified by Schultze in 1865 (4), but its functions in hemostasis and thrombosis weren't elucidated until 1881 by Bizzozero (5, 6). In 1906, Wright established megakaryocytes (MKs) in the bone marrow to be the source of platelets (7), though recent studies have shown that mature megakaryocytes in the lungs can also release platelets into the pulmonary vasculature (8). MKs produce billions of platelets daily through fragmentation, in which small cytoplasmic pieces bud off the megakaryocyte to become platelets (9). During fragmentation, MKs undergo a series of elongations to form proplatelet shafts, or cytoplasmic extensions which serve as assembly lines for platelet formation (10). Platelet-sized swellings then form along the shaft (11); as these platelets develop, they are loaded with the necessary organelles and granules from the MK parent (9). Platelets are equipped with mitochondria, a cytoskeleton, and a dense tubular system (DTS) (3, 12). Additionally, platelets contain secretory organelles categorized as alpha, dense, and lysosomal granules, which are transported and discharged by a surface-connected open canalicular system (OCS) (13). Dense granules generally contain small molecules such as ADP or serotonin, whereas alpha granules contain hemostatic factors such as fibrinogen, as well as other growth factors and cytokines (14). Upon complete generation, platelets are released from the bone marrow into circulation, where they live for the next 7–10 days (15). Historically disregarded as “cellular dust” (16), platelets have only recently emerged as having more diverse homeostatic processes including wound healing, angiogenesis, immunoregulation, and inflammatory response all key components to a stress response (2, 17–19).

## Platelet Function Beyond Hemostasisy Figure 1

Platelets are primarily responsible for the maintenance of normal hemostasis by the prevention of hemorrhage during vascular injury (20). Hemostasis is achieved by a careful balance of platelet interactions with vascular components, cytokine mediators, fibrinolytic agents, and plasma coagulation mechanisms (21). They assist in initiating a vascular response leading to vasoconstriction, and formation of a hemostatic plug (through adhesion, activation, and aggregation). The blood coagulation cascade is then initiated with expansion of the thrombus and massive release of platelet contents (22). The released factors then also assist in promoting tissue repair and resolving the repair process (23). The role of platelets in thrombosis is essential, and increasingly becoming well-understood. Given the complex content within platelets, researchers have recently begun to investigate platelet function beyond coagulation, and have implicated platelets in several processes including immunoregulation, infection, inflammation, and the pathogenesis of a growing list of diseases (neurodegenerative diseases, cardiovascular disease and cancer) (24–26). In the absence of a nucleus, the role of the platelet mitochondria in these processes has become a focus of intense studies, including how platelet dysfunction is associated with, contribute to, is affected by the disease pathologies (25).

**Figure 1:**
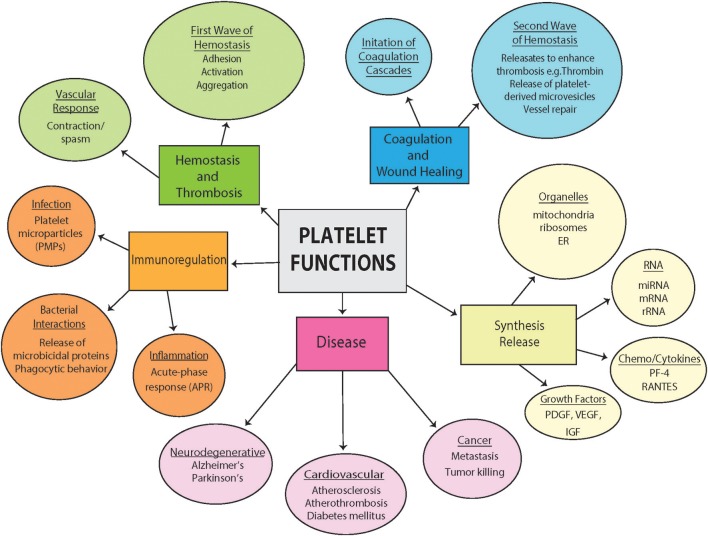
Diversity of platelet function. Highlighted are some of the diverse pathophysiological functions of platelets both in health and disease from hemostasis and thrombosis to contributions to disease. Included are also a section outlining diverse synthesis and release of platelets and important involvement in immunoregulation.

## Platelets: A Nucleus-Free Zone

Notably, mammalian platelets do not contain a nucleus (27). Interestingly, non-mammalian vertebrates have nucleated thrombocytes that have limited responses, to thrombin but not to ADP, serotonin or epinephrine (28, 29). As described (9), upon fragmentation, mature mammalian MKs segregate into anucleate platelets; thus, platelets are not endowed with the genomic genetic material generally considered a requirement for complex cellular function (16). Nuclear material (genomic DNA) generally provides functional autonomy; any needed protein can be transcribed from the genomic road map provided in the nucleus (19). However, the presence of a nucleus in platelets may hinder many of the important roles played by the platelet, at the expense of functional autonomy. To fulfill the many emerging functions, platelets need to be small (able to circulate in small vessels), flexible, highly efficient (produces proteins rapidly and efficiently) and highly sensitive (responds to stressors rapidly).

A human cell nucleus on average is much larger than a platelet (nucleus ~6 μm in diameter) (30), dictated by DNA content (3 billion base pairs in humans), and cytoplasmic factors (31). The presence of a nucleus even if small and compact, would greatly enhance a platelet's size thus reducing its ability to travel through small vessels and spaces. In addition to small size, platelets need to be flexible, capable of modulating their internal space to undergo extreme morphological changes (19), again allowing platelets to travel through even the smallest vessels in the circulatory system and enter tissues when needed. Further, this enhanced flexibility allows the platelet OCS to fill internal vacancies during activation to increase surface area available for interaction with blood plasma (19, 32). The presence of a nucleus in avian thrombocytes (nucleated platelets) makes them larger than mammalian platelets, causes them to spread less efficiently on collagen, and express much lower levels of the α_2b_β_3_ integrin required for aggregate formation (28). Similarly, the lack of a nucleus within the red blood cells (RBC), allows them to maintain their distinct bi-concave shape; but additionally, removes the need to maintain nuclear function and genome, allowing the RBC to focus on producing and maintaining hemoglobin. Platelets, like RBCs, do not need to regulate the health of a large complex nucleus, with its transcriptional machinery and chromosomes. Platelets can thus focus on what is needed for their roles in hemostasis and homeostasis with prepackaged, carefully selected RNA and translational machinery (without requiring transcriptional regulation of complex genomic DNA) (33, 34). Each platelet is equipped with an abundance of needed genetic information, and processing machinery required for a highly efficient rapid response (35), within minutes (required for hemostasis), rather than hours or days. Thus, based upon the literature and the lower efficiency of nucleated avian thrombocytes, we believe that the lack of a nucleus allows for improved platelet functional efficiency. More studies are required to support this notion. Interestingly, in the absence of a nuclear source of RNA, platelets are capable of taking up RNA material from external sources through microvesicles (MVs) (36). RNAs can also be donated from platelets to other cells through microvesicle mediated intercellular crosstalk (37–39). The microRNAs within these MVs have proven to be increasingly relevant to understanding the role of platelets in thrombosis, immune response, and various diseases. Not only miRNA but transcription factors and mitochondria are conveyed to such cells as neutrophils, mediated by 12-lipoxygenase and phospholipase A2-IIA (40). Thus, platelets have proven to be “intelligent” even in the absence of a nucleus: a platelet's ability to interact with their environment and efficiently respond to the needs of that environment, has marked them as far more complex than previously thought (41).

## Platelet Mitochondria Are Key to Function and Survival of Platelets Figure 2

To maintain the ability to rapidly respond to stressors or blood vessel damage (thrombosis), a highly efficient source of energy and metabolites are needed to orchestrate the response. Distinct from RBCs that are also devoid of mitochondria, platelets are equipped with mitochondria (42). Interestingly, mitochondria have a number of features in common with a nucleus, both contain DNA, both are surrounded by a double plasma membrane, and both can divide during the cell cycle (30). However, the role of the mitochondria, referred as the “powerhouse of the cell,” is quite different, playing essential roles in energy production and metabolism (43). The mitochondria is home to key energetic processes such as the tricarboxylic acid (TCA) cycle and oxidative phosphorylation (OXPHOS), both of which are involved in the production of adenosine triphosphate (ATP) (44). However, studies have implicated mitochondria in many processes beyond energy production, such as the generation of reactive oxygen species (ROS) (45), Ca^2+^ homeostasis (46), apoptosis regulation (47), and ER-stress response mechanisms (48). Mitochondrial health and dysfunction also appear to be involved in aging (49), as well as neurodegenerative diseases (e.g., Alzheimer's (50) and Parkinson's disease (51). Healthy platelets contain between 5 and 8 mitochondria, the majority of which must remain uncompromised for the platelet to maintain proper function. In healthy platelets, mitochondria has been demonstrated to serve a variety of purposes as described for nucleated cells, from metabolism, activation, ATP production to the regulation of cell processes and viability (42, 52).

**Figure 2:**
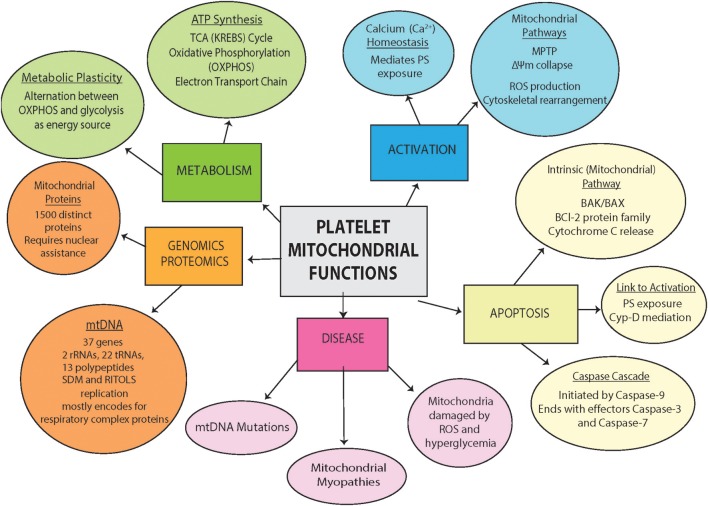
Platelet mitochondrial Functions. Outlined are platelet mitochondria contents (genomics and proteomics), physiological function (metabolism), and involvement in pathology and disease (process of activation, apoptosis and disease involvement).

In the absence of nuclear control, platelet health is largely determined by the health of their mitochondria (53). As is apparent in a number of diseases, an excess of damaged platelets can lead to premature apoptosis; therefore, it is essential to keep platelet mitochondria in good health (25). The turnover rate for mitochondria in various nucleated cells ranges from 9 to 24 days (54, 55). Considering, the lifespan of a platelet (7–10 days), the necessity of mitochondria for energy production, and the inability to consistently replenish nuclear encoded mitochondria proteins, the lifespan of the platelet mitochondria likely determines the platelet lifespan. A mitochondrial protein Bcl-xl, master regulator of mitochondrial apoptosis has been reported to determine platelet lifespan (56). Moreover, a recent study implicated TNF-alpha-driven megakaryocyte reprogramming leading to increased mitochondrial mass and activity as being a major contributor to the observed hyperactivity and thrombosis associated with aging (57). Multiple factors affect mitochondrial health and some of the key components determining mitochondria function and lifespan, especially in context of the anucleate platelets, will now be discussed.

### Mitochondria DNA (mtDNA)

A complete mitochondrial genome is needed for both proper mitochondrial as well as platelet function, and the prevention of a number of mitochondrial related diseases such as mitochondrial myopathies caused by mtDNA mutations (58). Mitochondria, along with chloroplasts in plants, are the only organelles besides the nucleus to contain genetic material (59). Human mitochondrial DNA (mtDNA) is double-stranded, circular, and relatively small: at 16.6 kbp, human mtDNA is comprised of 37 genes encoding two rRNAs, 22 tRNAs, and 13 polypeptides, all of which are components of the OXPHOS enzyme complexes (60). Similar to bacterial chromosomal DNA, mtDNA is organized, in multiple copies, in nucleoids within the mitochondria (61). Because they likely originated from prokaryotic ancestors, mitochondria are largely self-sufficient: they are able to maintain, transcribe and translate mtDNA internally and independently (62). Mitochondria have several modes of mtDNA replication which differ significantly from the nuclear mode of replication including the strand-displacement mode (SDM) (63, 64), ribonucleotide incorporated throughout the lagging strand replication (RITOLS) (65) and coupled leading-lagging strand synthesis (66). mtDNA copy number is considered an indirect measure of mitochondrial function (67) and its quantification in peripheral blood, majorly reflects the mtDNA copy number in leukocytes and platelets (68). The epigenetic regulation of platelet mtDNA is of particular importance with higher methylation of platelet mtDNA being a possible biomarker for cardiovascular disease (CVD) (69, 70).

### Mitochondrial Functions

In addition to mtDNA, mitochondria are equipped with a complex array of proteins offering significant insights into mitochondrial activity (71). Mitochondria contain around 1,500 distinct proteins in mammals, compared to around 1,000 in yeast (72). Since the majority of the proteins encoded by mtDNA form components of the respiratory chain complexes, most mitochondrial proteins are encoded by the nucleus and imported into the mitochondria from the cytosol with the help of mitochondrial translocases (73). Each protein is coupled with a distinct import signal which guides it to the appropriate mitochondrial membrane, to which it is then inserted: outer membrane proteins are integrated by the TOM complex, whereas TIM23 is the presequence translocase responsible for inner membrane protein integration (74). The mechanism by which proteins are incorporated into the mitochondria is posttranslational: unlike ER-protein import, mitochondrial proteins are synthesized in the cytosol as precursor proteins before being translocated into a mitochondrion (75).

#### Mitochondria and Energy Metabolism in the Platelets

While most human nucleated cells contain hundreds, if not thousands, of active mitochondria, platelets generally contain only 5–8 mitochondria per cell (42). However, metabolically, platelets are quite active: for example, compared to resting mammalian muscle cells, platelets have much higher levels of ATP-turnover (69). This energy demand is met using a metabolic system which combines the efforts of glycolysis and mitochondrial OXPHOS. In platelets, glycolysis provides about 60% of cellular ATP, while OXPHOS provides the remaining 30–40% (25). Out of the platelet's total mitochondrial function, 50% is dedicated to ATP production; the reserve energy is responsible for, among other activities, cellular response to oxidative stress (76). ATP is essential to proper platelet function: several key processes that occur within the platelet, such as the maintenance of calcium homeostasis, require a constant energy supply. Interestingly, platelets have been shown to have a metabolic flexibility that helps them meet this energy demand; activated platelets exhibit a glycolytic phenotype even as they preserve mitochondria function (77). This ability to utilize glycolysis or fatty acid catabolism instead of OXPHOS (mitochondrial ATP production) allows the platelet to adapt to different situations, such as hypoxia or the presence of mitochondrial inhibitory agents (78). Several studies have shown that platelet aggregation along with other metabolic activities are only fully interrupted when mitochondrial OXPHOS and glycolysis are inhibited simultaneously (79). Indeed, double knockout of GLUT1 and GLUT3 (major transporters of platelet glucose) leads to mitochondria reprograming, reduced thrombosis and reduced platelet activation along with thrombocytopenia (80). This suggests that this metabolic plasticity is the key to enabling platelets to meet their extraordinary energy demand with so few mitochondria.

#### Mitochondria and Platelet Activation

Platelets are activated during the adhesion events of primary homeostasis, and the initiation of the blood coagulation cascades (81). Until recently, it was assumed that the only role mitochondria played in this process was an energetic one (78). However, new studies have demonstrated the contributions of several mitochondrial functions in platelet activation such as the mitochondrial permeability transition (MPT) (82, 83), increased ROS generation (84–86), and collapse of mitochondrial membrane potential (ΔΨ_m_). Platelet activation is mediated by several agonists: collagen, thrombin, and ADP have all been implicated in the regulation of hemostasis (87). The activity of these agonists is mediated by a common increase in intracellular calcium (88). Mitochondria do little to regulate platelet calcium levels (89), but a simultaneous increase in intramitochondrial calcium levels does mediate phosphatidylserine (PS) exposure, without affecting integrin activation and granule release (86). Increased mitochondrial calcium levels also correlate with mitochondrial ROS imbalance and MPT pore activation (90). Strong platelet activation characterized by the drastic increase in mitochondrial and cytosolic calcium also seems to initiate the collapse of the mitochondrial membrane potential (ΔΨ_m_) via a cyclophilin D (CypD)-dependent mechanism (56). Thus, this collapse, mediated by mitochondrial calcium, contributes to further ROS generation and the initiation of the PS exposure essential for platelet adhesion (85). Interestingly, these mitochondrial activation pathways also contribute to the platelet apoptosis framework (84, 91, 92).

#### Mitochondria and Apoptosis Figure 3

Long associated only with nucleated cells, apoptosis is a mechanism of systematic cell deletion which can be induced or inhibited by both normal and abnormal stimuli (96). However, recent studies have identified apoptosis in the anucleate platelet (96, 97). Morphologically, platelet apoptosis is characterized by blebbing, platelet shrinkage, PS exposure, fragmentation into microparticles, and filopod formation (96). Apoptosis follows either an extrinsic or intrinsic pathway, the former stimulated by the activation of cell-surface death receptors, and the latter mediated by mitochondrial coordination of pro- and anti-apoptotic members of the Bcl-2 family (98). The presence of Bcl-2 family proteins within platelets, along with the platelet PS exposure characteristic of apoptosis, suggests that platelets might primarily follow an intrinsic apoptotic pathway (99, 100). Bcl-x_L_, the key regulator of platelet survival, is responsible for inhibiting BAK and BAX, two pro-apoptotic proteins which serve to damage the mitochondria: studies in which either Bcl-x_L_ or BAK/BAX were impaired saw interference with natural platelet lifespan (98, 101, 102). Overexpression of Bcl-2 family pro-survival proteins can increase survival of platelets in the circulation (103). However, deletion of Bcl-2 in mice did not affect thrombopoiesis or platelet life span (104), supporting compensatory responses. When platelets come under stress or reach their natural end, the survival signal is overwhelmed and causes the activation of BAK and BAX, initiating the subsequent release of mitochondrial components such as cytochrome *c* through pores in the mitochondrial membrane (105). The presence of cytochrome *c* in the cytosol triggers the apoptotic caspase cascade, which begins with the initiator caspase-9 and ends with the effectors caspase-3 and caspase-7, which cleave hundreds of intracellular components and effectively destroy several essential cellular processes (106). Interestingly BCL2 family proteins are also involved in platelet formation with the anti-apoptotic family member BCL2L2 being involved in increasing megakaryocyte proplatelet formation in cultures of human cord blood (107).

**Figure 3:**
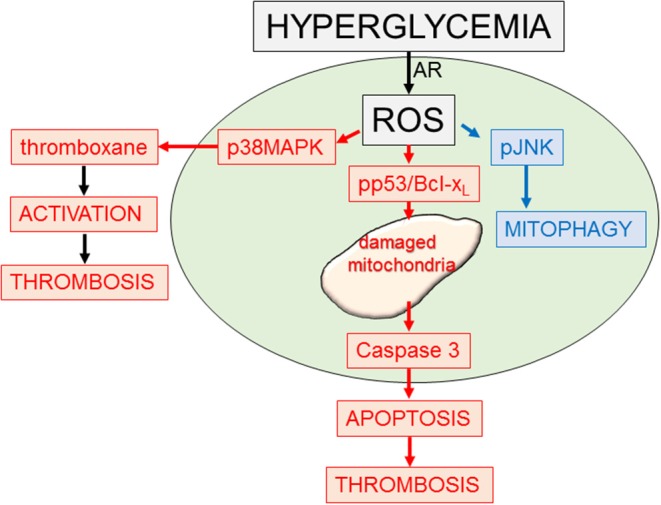
Platelet response to hyperglycemia. Diagram outlining some of the signaling and functional responses to stress (hyperglycemia). Increased glucose (hyperglycemia) through the aldose reductase enzymic system can lead to enhanced reactive oxygen species (ROS). This can activate multiple pathways including p38MAPK, promoting platelet activation and thrombosis (93); phosphor-p53 and Bcl-xl, promoting mitochondrial damage, apoptosis, and thrombosis (94); and a mitophagy rescue response, removing toxic damaged mitochondria (95).

#### Mitophagy in Platelets/Mitochondrial Turnover in Platelets Figure 3

Highlighting the importance of protecting platelet mitochondria in maintaining platelet health and lifespan, the protective process of induced mitophagy was recently described in platelets (95). This is distinct from basal platelet autophagy needed for an important role in platelet activation (108–110). Mitophagy can be generated by Parkin-independent or -dependent pathways (111–115) The process in platelets is Parkin-dependent and protects the platelet from oxidative stress and mitochondrial mediated damage (95). In the absence of a nucleus the platelet requires prepacking of the highly ordered and complex process, from initial phagophore formation (nucleation) to subsequent expansion of the membrane by ubiquitin-like conjugating systems, microtubule-associated protein 1 light chain 3 (LC3), and the autophagy protein system (ATGs), ultimately the phagophore completely surrounding its target, followed by fusion with a lysosome, leading to content degradation by lysosomal enzymes (116–118). The energy required to sort and prepackage the mRNA components for this process in anticipation of mitochondria stress/damage suggests an essential role for platelet mitochondria in health and disease. Platelet mitophagy removes toxic damaged mitochondria (as in diabetes mellitus) preventing platelet apoptosis (95, 119). If platelet mitophagy is impaired, increased platelet apoptosis can contribute to enhanced thrombosis (95).

## Platelet Mitochondria in Disease

Because mitochondria play such an integral role in platelet metabolism, activation, and apoptosis, it is no surprise that mitochondrial dysfunction contribute to dysfunctional platelet activity and apoptosis in several diseases, most notably Alzheimer's and Parkinson's (120), cardiovascular disease (CVD) (121), diabetes mellitus (122), and sepsis (123). Apoptotic platelets induce clotting 50–100 times faster than normal platelets, because phosphatidylserine on the platelet surface acts as a catalytic site for clotting enzyme assembly and thrombin generation (124, 125). Recent studies have therefore provided invaluable insight into the complex mitochondrial mechanisms that determine platelet function in relation to tissue homeostasis (126). Due to the decreased fidelity of PolyG during replication, the mutation rate of mtDNA is about 100-fold higher than that of nuclear DNA (127). Additionally, the process of mitochondrial DNA segregation occurs randomly and with far less organization than in the nucleus, sometimes leaving daughter cells with similar, but not identical, copies of mtDNA (128). Combined, there is increased risk for mtDNA abnormalities and mutations that can have severe health consequences, hindering ATP generation and increasing oxidative stress (129). Mutations which impact mitochondrial functionality are also relevant in aging (130) and age-related diseases, such as diabetes mellitus (58), Parkinson's (51), and cardiovascular disease (69). Platelet mitochondrial DNA in the circulation may serve as biomarkers for disease (131–133).

Cardiovascular diseases (CVD) including atherosclerosis and thrombosis are the leading causes of death for patients with diabetes (134). Diabetes mellitus, characterized by acute and chronic hyperglycemia have been shown to increase mitochondrial ROS production in platelets leading to activation (85, 93, 135). Platelets have also been identified as leading players in the development of atherosclerotic lesions, contributing, along with monocytes, to the inflammatory environment of atherosclerosis (136, 137). Type 2 diabetes results in alterations of platelet ATP production with an initial increase in platelet mitochondrial ATP content and platelet activity (93, 138), followed by platelet apoptosis and a decrease in ATP production, in the presence of severe persistent oxidative stress (94, 138, 139). Antiplatelet therapies, as well as hyperglycemic control treatments, have become increasingly relevant for the regulation of the high levels of platelet reactivity observed in Type 2 diabetes (140, 141). Preserving platelet mitochondrial function may be an additional means of decreasing the risk of potentially fatal thrombotic events for diabetic patients (142). Platelets may also serve essential functions in immunoregulation (143–146). Alterations in the bioenergetics of platelet mitochondria have been observed in cases of sepsis (147), contributing to drastic, but impermanent, organ failure (148). Thrombocytopenia is associated with increased mortality in septic shock (149, 150). Recently, platelet mitochondria have also been reported to incite an inflammatory response upon activation through release of bioenergetically active mitochondria in free as well as encapsulated form (151). Interestingly, the alphaproteobacterium *Rickettsia prowazekii* may be an evolutionary ancestor of the mitochondria (152).

Both Parkinson's and Alzheimer's are severe neurodegenerative diseases that have been linked to platelets, mitochondrial dysfunction and platelet apoptosis (153, 154). Platelets, are considered to be structurally and functionally similar to neurons and have been shown to rich in key proteins associated with the neurons and brain (155–157). Indeed, after the brain, platelets contain the highest amounts of Amyloid precursor protein, synuclein and tau, and are major contributors to the circulating levels of these key proteins involved in neurodegenerative diseases (158). As in the other diseases profiled, Alzheimer's presents a compromised mitochondrial ETC; in particular, the activity of cytochrome c oxidase, an oxidative metabolic component of Complex IV, has been shown to be impaired (159). Platelet mitochondria in Alzheimer's have been observed as having increased levels of oxidative stress leading to mitochondrial damage and platelet apoptosis (160). Studies of mitochondrial function in Parkinson's disease have identified mutations or defects in Complex I-IV of the ETC, α-synuclein, PARKIN, PINK1, DJ-1, and LRRK2 (139). A decrease in Complexes I and IV can develop quickly within the first year of Parkinson's (161, 162), and low levels of activity in platelet mitochondrial complexes I and II/III in early, untreated Parkinson's (163). Other key protein associations include, NADH CoQ reductase, key to Complex I dysfunction (154), coenzyme Q10, a key electron receptor in Complexes I and II of the mitochondrial ETC (164–166) and the neurotransmitter-degrading enzyme monoamine oxidase B (MAO) (167–169). Implicated in the pathogenesis of both Alzheimer's and aging in general, platelet MAO has been used as a peripheral biomarker for the onset of Alzheimer's and Parkinson's (170, 171). Also consistent with the association of oxidative stress induced mitochondrial damage and apoptosis with Parkinson's disease, levels of the oxidative protective protein methionine sulfoxide reductase type 2 (Msrb2) was recently shown to be reduced in platelets of Parkinson's disease patients leading to increased platelet apoptosis (119). Indeed, platelet mitochondria may serve as an important biomarker in PD. These neurodegenerative diseases once again demonstrate the essential nature of platelet mitochondria for survival as reduced function and damage either through genetic defects or environmental stress leads to apoptosis and premature platelet death.

With the importance of mitochondria in platelet function and the potential contributions of mitochondria dysfunction to aging (49, 130) and age-related diseases, such as diabetes mellitus (58, 93, 94), Parkinson's (51, 119), and cardiovascular disease (69), targeting platelet mitochondria may serve as adjunct therapies. Drugs targeting platelet mitochondria metabolism and apoptosis may help prevent pathological thrombosis and contributions to disease. The genomics and proteomics of the mitochondria (Figure 2) provide multiple potential targets as outlined in an excellent recent review by Fuentes et al. (172). However, selective targeting to platelets may require further coupling to platelet targeting agents.

## Conclusion

Mitochondria in nucleated cells have been well-described. While mitochondria functions are similar, its role becomes increasingly important in the nucleus-free zone of the platelet. As described in this review, not only are mitochondria involved in energy metabolism and ATP production in the platelets, they are also the central drivers of platelet activation and apoptosis; both events critical for platelet function and lifespan. The pathophysiological role played by platelet and their mitochondria in many systemic diseases remain under intense investigation. Therapies targeting platelet mitochondria may ultimately prove beneficial for such disease processes.

## Author Contributions

HM and JH conceived the paper. HM wrote the first draft. KJ, TT, and JH extensively reviewed and edited the paper.

### Conflict of Interest

The authors declare that the research was conducted in the absence of any commercial or financial relationships that could be construed as a potential conflict of interest.
